# Lethal and Sublethal Toxicity of Beta-Carboline Alkaloids from *Peganum harmala* (L.) against *Aedes albopictus* Larvae (Diptera: Culicidae)

**DOI:** 10.3390/toxics11040341

**Published:** 2023-04-03

**Authors:** Nan Jiang, Li Chen, Jinmei Li, Wenyong Li, Shuanglin Jiang

**Affiliations:** 1 Key Laboratory of Environmental Medicine Engineering, Ministry of Education, School of Public Health, Southeast University, Nanjing 210009, China; 2 School of Biology and Food Engineering, Fuyang Normal University, Fuyang 236037, China

**Keywords:** larval toxicity, lethal and sublethal effects, total alkaloid extracts, beta-carboline alkaloids, *Peganum harmala* (L.), *Aedes albopictus*

## Abstract

Plant-derived agents are powerful bio-pesticides for the eco-friendly control of mosquito vectors and other blood-sucking arthropods. The larval toxicity of beta-carboline alkaloids against the Asian tiger mosquito, *Aedes albopictus* (Skuse) (Diptera: Culicidae), was investigated under laboratory conditions. The total alkaloid extracts (TAEs) and beta-carboline alkaloids (harmaline, harmine, harmalol, and harman) from *Peganum harmala* seeds were isolated and tested in this bioassay. All alkaloids were tested either individually or as binary mixtures, using the co-toxicity coefficient (CTC) and Abbott’s formula analysis. The results revealed considerable toxicity of the tested alkaloids against *A. albopictus* larvae. When all larval instars were exposed to the TAEs at 48 h post-treatment, the mortality of all larval instars varied in a concentration-dependent manner. The second-instar larvae were the most susceptible to different concentrations of TAEs, and the fourth-instar larvae were more tolerant to TAEs than the second-instar larvae. Especially, the third-instar larvae exposed to all alkaloids also showed that all doses resulted in an increased mortality of the third-instar larvae at 48 h post-treatment, and the toxicities of the tested alkaloids in a descending order were TAEs > harmaline > harmine > harmalol, with the LC_50_ values of 44.54 ± 2.56, 55.51 ± 3.01, 93.67 ± 4.53, and 117.87 ± 5.61 μg/mL at 48 h post-treatment, respectively. In addition, all compounds were also tested individually or in a 1:1 ratio (dose LC_25_/LC_25_) as binary mixtures to assess the synergistic toxicity of these binary combinations against the third-instar larvae at 24 and 48 h post-treatment, respectively. The results demonstrated that when tested as a binary mixture, all compounds (especially TAEs, harmaline, and harmine) showed their synergistic effects, exceeding the toxicity of each compound alone. Interestingly, the obtained data further revealed that the TAEs at sublethal doses (LC_10_ and LC_25_) could significantly delay the larval development and decrease the pupation and emergence rates of *A. albopictus*. This phenomenon could be helpful in order to develop more effective control strategies for different notorious vector mosquitoes.

## 1. Introduction

*Aedes albopictus* (Skuse) (Diptera: Culicidae), commonly known as the Asian tiger mosquito, is an aggressive daytime human-biting behavior and one of the most important nuisance mosquito species in the world [1]. *A. albopictus* has proven to be an infectious vector, which can transmit a spectrum of at least 23 human pathogens causing various diseases including dengue virus, West Nile virus, and chikungunya virus [2] (Manni et al., 2017). The World Health Organization (WHO) estimates that there are 68,000 clinical cases each year, but more than 3 billion people in Asia are at risk of exposure [3,4]. It is well known that one way to reduce the mosquito populations is targeting mosquito larvae with chemical insecticides, such as pyrethroids, organophosphates, and insect growth regulators [5,6]. However, repeated use of these chemical insecticides can lead to the development of resistance in mosquitoes or affect human health, or to undesirable effects on non-target organisms [7]. For these reasons, there is an urgent need to develop new insecticides which are more environmentally safe and also biodegradable and are targeted specifically against vector mosquitoes. In fact, plant extracts and plant-derived compounds belonging to many families have been reported to possess larvicidal properties against *Aedes*, *Culex*, and *Anopheles* mosquitoes (Diptera: Culicidae) [8,9].

*Peganum harmala* L. (Zygophyllaceae) is one of the most famous plants, the so-called Harmal, used in popular medicine. As a perennial herb, it is widely distributed in Europe, North Africa, Middle East, Central Asia, Northwest India, and Northwest China [10,11]. The full plant, aerial parts, seeds, and fruits have generally been reported to exhibit a traditional herbal medicine with various pharmacological activities, such as analgesic activity, antiproliferative, anti-parasitological, and antimicrobial activities, anticancer, and other activities [10,12]. The seeds of *P. harmala* have also been included in the Drug Standards of the Ministry of Public Health of the People’s Republic of China, and the therapeutic dose of them is 4~8 g [13]. Ulteriorly, the quality standards for the seeds and the total alkaloid extracts from the seeds of *P. harmala* have been formulated in previous studies [14]. The seeds of *P. harmala* contain about 2% and 6% (*w*/*w*) pharmacologically active alkaloids, which are mostly beta-carbolines such as harmaline, harmine, harmalol, and harmane. In particular, the total content of harmine and harmaline is more than 50% in the total alkaloid extracts (TAEs) of *P. harmalala* seeds [12,15]. Modern pharmacology has also revealed that *P. harmala* alkaloids can inhibit monoamine oxidase A (MAO-A), acetylcholinesterase (AChE), and butyrylcholinesterase (BChE), and interact with γ-aminobutyric acid (GABA), and induce apoptosis and DNA damage [11,12,16,17].

There have been some reports about the pesticidal bioactivity of TAEs and beta-carboline alkaloids, indicating their lethal and sublethal effects on many pests. We previously evaluated the insecticidal activity of TAEs and its alkaloids against a variety of pests in laboratory and field conditions [18,19,20]. Several studies have also reported on the effectiveness of alkaloid extracts of *P. harmala* against many insects and parasites [21,22,23,24]. In addition to lethal effects, several studies have also revealed that *P. harmala* total alkaloids or its beta-carboline alkaloids could induce sublethal effects on insect development, reproduction, lifespan, and behavior, even at low-dose levels [25,26,27,28,29,30]. Therefore, these results demonstrated that TAEs and its beta-carboline alkaloids have a variety of unique insecticidal activities against pests, indicating that these alkaloids are potential plant-derived agents. 

Synergistic effects between different chemical components in plants have been documented, with the toxicity or other bioactivity of one compound significantly increased in the presence of other compounds [9,30,31]. In fact, the optimal efficacy of a medicinal plant may not be due to one major active component, but to the combined effect of distinct compounds originally in the plant. For instance, the antibacterial and antifungal activities of beta-carboline alkaloids of *P. harmala* seeds were increased when tested as binary or total alkaloidal mixtures showing a kind of synergism among these alkaloids such as harmaline, harmine, harmane, and harmalol [32]. Another study also obtained similar results on the combined toxic effects of two alkaloids, harmine and ricinine, on beet armyworm (Lepidoptera: Noctuidae) [33]. Because such responses are likely to occur affecting the total alkaloid extract of *P. harmala* seeds, it is important to evaluate an active synergistic mixture with the pesticide properties. Consequently, it could be concluded that *P. harmala* alkaloids have potential in the search for new and eco-friendly pesticidal agents for the control of the vector mosquitoes such as *A. albopictus*. 

To the best of our knowledge, the insecticidal activity of harmala alkaloids against vector mosquitoes has not been reported. Therefore, this study aims to evaluate the lethal and sublethal toxicity of beta-carboline alkaloids from *P. harmala* against *A. albopictus* larvae, explore the synergistic toxicity of binary mixtures of four isolated alkaloids at low-dose levels, and provide a reference for the further exploration and utilization of the plant as a botanical insecticide.

## 2. Materials and Methods

### 2.1. Material, Standards, and Reagents

**Figure 1:**
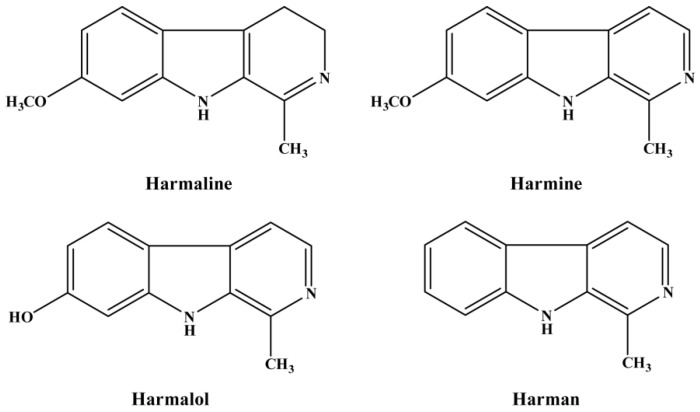
Molecular structural formulas off our isolated alkaloids (harmaline, harmine, harmalol, and harman) from *Peganum harmala* seeds.

*P. harmala* L. fresh seeds were collected in August 2020 from ripened samples at Huan in County Northwest China and were dried to a constant weight at 60 °C. The raw material was identified by Prof. Xiaoqiang Guo, Life Science and Technology College of Longdong University, China. Harmaline, harman, harmalol, and harmine with high (>95%) label purities were purchased from Fluka Chemical Co. and used as reference materials. The molecular structural formulas of the four alkaloids are shown in Figure 1.

### 2.2. Total Alkaloid Extracts of P. harmala Seeds

The total alkaloid extracts of *P. harmala* seeds were extracted by the method we previously described [20]. Specifically, dried and powdered seeds (1 kg) were macerated four times with 1 L of methanol for 24 h. The extracts were combined and then the solvent was evaporated to dryness, using a rotary evaporator. The methanol extract of the seeds was successfully separated using hexane and dichloromethane in 10% NH_4_OH medium (pH 9–10). The obtained fractions were concentrated, yielding the total alkaloid extracts (TAEs) of *P. harmala* seeds [14]. Fractionation of the individual alkaloids was prepared according to the method of Kartal et al. (2003) [34]. Briefly, the TAEs were eluted on a silica gel column initially with pure CHCl_3_ and increasing amounts of CH_3_OH. All fractions obtained were tested on precoated TLC plates using CHCl_3_/CH_3_OH (9:1, *v*/*v*). Similar fractions were combined and washed sequentially in CHCl_3_ and CH_3_OH. The crystallized samples were compared by their retention times on high-performance liquid chromatography (Agilent, Santa Clara, CA, USA, 1290 Infinity II) relative to authentic samples, and using electrospray mass spectrometry (Thermo Scientific, Waltham, MA, USA, Orbitrap Exploris™ 240) [15]. All fractions obtained were dried and stored in closed dried bottles for further bioassays.

### 2.3. Insect

*A. albopictus*, maintained for more than 100 generations without exposure to any known insecticide, was obtained from laboratory colonies in the State Key Laboratory of Pathogen and Biosecurity, Institute of Microbiology and Epidemiology, Beijing. According to the World Health Organization’s standards for larval susceptibility test methods [35], eggs for the study were obtained by feeding mated 15 generation females provided with a 10% sucrose solution for 12 h, and then provided with a rat placed in resting cages (25 × 25 × 35 cm) overnight for blood feeding to be carried by the females. All rats were maintained and used in accordance with the Guidelines of the Animal Care and Use Committee of Fuyang Normal University (license number 083/2021). Larvae were fed a diet of dog biscuits, milk powder, beef liver, and yeast powder in a ratio of 2:1:1:1, respectively. The insectary room was maintained at a photoperiod of 14:10 (L/D) h, temperature of 27 ± 1 °C, and relative humidity of 75–85%.

### 2.4. Evaluation of Bioefficacy

#### 2.4.1. Individual Toxicity of the Tested Alkaloids

The larvicidal activity of TAEs and the four major compounds, namely harmaline, harmine, harmane, and harmalol, isolated from *P. harmala* seeds was evaluated according to the WHO protocol [35] (2005). Based on previous pre-experiments, each compound was tested at 20, 40, 60, 80, 100, and 120 μg/mL. Stock dilution (*w*/*v*) of each material was dissolved in 1 mL of methanol and then diluted in 249 mL of distilled water to obtain each of the required concentrations. The control was prepared using 1 mL methanol in 249 mLof distilled water. Thirty required larvae were introduced into each solution. Five biological replicates were then performed for each concentration, with 30 larvae per replicate (*n* = 150). Larval mortality was recorded at 24 h and 48 h post-treatment and corrected according to Abbott’s formula as Corrected mortality (%) = [(Treated mortality—Control mortality)]/(100-Control mortality)] × 100. The 25 and 50% lethal concentrations (LC_25_ and LC_50_, respectively) were evaluated by probit analysis [36].

#### 2.4.2. Synergistic Toxicity of Binary Mixtures of Four Isolated Alkaloids

In this bioassay, four isolated alkaloids (harmaline, harmine, harmalol, and harman) were combined in a 1:1 ratio (doses LC_25_/LC_25_), binary combinations of the alkaloids were assessed for the third-instar larvae, and TAEs as the positive control group. Larval mortality was recorded at 48 h post-treatment and corrected using Abbott’s formula. The co-toxicity coefficient (CTC) of the mixtures was calculated according to Alonso-Amelot and Calcagno [37] (2000) as follows:

CTC = [(*A* + *B*)_exper_/(*A* + *B*)_theor_] × 100.

where

(*A* + *B*)_exper_ = Sum of experimental (observed) mortalities of *A* + *B.*

(*A* + *B*)_theor_ = Sum of experimental (expected) mortalities of *A* + *B.*

### 2.5. Sublethal Effects of the Tested Alkaloids on Larval Growth

In this experiment, TAEs were used to evaluate the sublethal effects. TheLC_10_ and LC_25_ values of the second-instar larvae determined by a bioassay at 48 h post-treatment were selected as the sublethal concentrations. The second-instar larvae were placed in a 250 mL glass beaker and exposed to the LC_10_ and LC_25_ doses for each test. More than 30 second-instar larvae were introduced into each concentration, and five biological replicates were performed for each concentration, with more than 30 larvae per replicate (*n* > 150) [35]; 48 h after treatment, dead larvae were counted and only alive larvae were transferred to glass beakers through a small filter, and larvae were provided with the larval food mixture at a concentration of 50 mg/L until pupation. Larval development was surveyed daily until all larvae had either pupated or died. Pupae from each treatment were removed daily and were transferred into a cup with distilled water until the adults emerged. The development of mosquito larvae, pupae, and adults emerging each day was recorded.

### 2.6. Statistical Analysis

The data from the larval mortality tests were subjected to an analysis of variance (ANOVA of square root transformed percentages). Differences between the treatments were determined by Tukey’s multiple range tests to compare differences at *p* < 0.05 significance level. The sublethal and lethal dosages of each material to the tested larvae were calculated by using probit analysis. All data were analyzed using the SPSS Statistical Software Package version 16.0. The results with *p* < 0.05 were considered statistically significant.

## 3. Results

### 3.1. Contents of the Isolated Alkaloids of P. harmala

According to the dry weight of seeds, the *w*/*w* extraction yield of the total alkaloid extracts (TAEs) of *P. harmalala* seeds, the TAE was 4.83% for hexane and dichloromethane. It was found that harmaline and harmine were the main alkaloids, with a content of 2.62% and 1.59% (*w*/*w*), respectively. And their contents were higher than that of the others (harmalol, 0.32% and harman, 0.15%). The chemical structures of these four major compounds are shown in Figure 1. Based on the literature, the total alkaloid content of *P. harmala* seeds varied between 2% and 6% [14,15], and the ratio of alkaloids in *P. harmala* may vary during different stages of growth. 

### 3.2. Larvicidal Activity of the Tested Alkaloids

**Figure 2:**
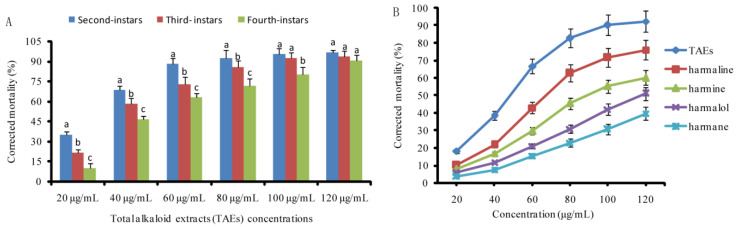
Individual toxicity of the tested alkaloids against *Aedesalbopictus* larvae. (**A**) Corrected mortality of the total alkaloids of *P. harmala* seeds (TAEs) against the second-, third- and fourth-instar larvae of *A. albopictus* at 48 h post-treatment. (**B**) Comparison of individual toxicity of pure alkaloids and TAEs against third-instar larvae of *A. albopictus* at 48 h post-treatment. Corrected mortality (±SE) followed by different letters in the same concentration in bars indicate significant difference (*p* < 0.05) according to Tukey’s test.

The total alkaloid extracts (TAEs) of *P. harmala* seeds exhibited a larvicidal effect against the second-instar larvae to fourth-instar larvae of *A. albopictus* at 48 h post-treatment, indicating that the mortality of all the larval instars varied in a concentration-dependent manner (Figure 2a). The second-instar larvae were the most susceptible to different concentrations of TAEs, and the fourth-instar larvae were more tolerant to TAEs than the second-instar larvae (*p* < 0.05). For example, the mortality rate of the second-instar larvae was over 80% at a 60 μg/mL concentration, while the mortality rate of the fourth-instar larvae was only over 80% at a 100 μg/mL concentration (Figure 2a). In this study, the third-instar larvae were thus used as toxicity assays unless otherwise specified. Furthermore, the third-instar larvae exposed to all alkaloids tested (TAEs and four compounds) also showed that all doses resulted in an increased mortality of the third-instar larvae at 48 h post-treatment, although harmalol and harman showed relatively low larvicidal activity. The larvicidal activity of the tested alkaloids in a descending order was TAEs > harmaline > harmine > harmalol > harman (Figure 2b). 

### 3.3. Comparison of the Lethal Concentration (LC_50_) of Third-Instar Larvae

**Figure 3:**
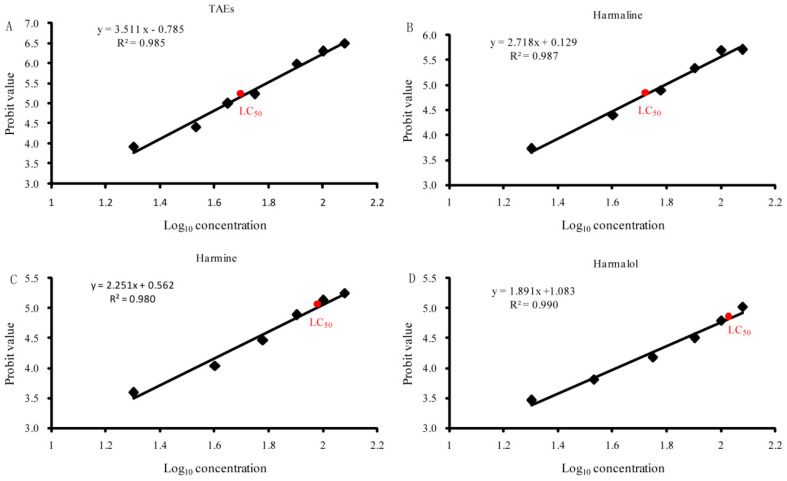
Evaluation of the median lethal concentration (LC_50_) of the tested alkaloids against third- instar larvae. Linear regression analysis of the log value of alkaloid concentrations and probit value of larval mortalities at 48 h post-treatment. (**A**) TAEs, (**B**) harmaline, (**C**) harmine, and (**D**) harmalol, with the LC_50_ values of 44.54 ± 2.56, 55.51 ± 3.01, 93.67 ± 4.53, and 117.87 ± 5.61 μg/mL, respectively. The LC_50_ value was calculated from the average of three replicates.

To further study the toxicity of the tested alkaloids (TAEs, harmaline, harmine, harmalol, and harman) to the third-instar larvae of *A. albopictus*, a bioassay was performed by the probit analysis method. The obtained results revealed that the toxicity of the third-instar larvae increased with the increasing concentrations (ranging from 20 to 120 μg/mL) of the tested alkaloids at 48 h post-exposure, as shown in Figure 3. There was a good linear relationship between the logarithm of each compound concentration and the probit value of the mortality for 48 h post-treatment. According to the obtained formula, the LC_50_ value of each compound was calculated. The results revealed the significant larvicidal effects of the tested alkaloids including TAEs, harmaline, harmine, harmalol, and harman, with the LC_50_ values of 44.54 ± 2.56, 55.51 ± 3.01, 93.67 ± 4.53, and 117.87 ± 5.61 μg/mL, respectively, while the LC_50_ value of harman exceeded the highest concentration tested (120 μg/mL).

### 3.4. Synergistic Toxicity of Binary Mixtures

Four isolated alkaloids (harmaline, harmine, harmalol, and harman) were tested individually or in a 1:1 ratio (dose LC_25_/LC_25_) as binary mixtures to assess the synergistic toxicity of these binary combinations against the third-instar larvae at 24 and 48 h post-treatment, as shown in Table 1. The results revealed that a considerable synergistic effect between all combinations was significantly increased when examined as binary mixtures than when each compound was examined individually. A mixture of harmaline and harmine at their LC_25_ against the third-instar larvae of *A. albopictus* resulted in 59.2% and 79.3% mortality at 24 and 48 h post-treatment, respectively. In addition, the binary mixtures of harmane with harmine or harmalol showed weak synergistic effects, respectively. Notably, TAEs also showed excellent activity against the third-instar larvae at 24 and 48 h after treatment. Thus, the results revealed that the activity of the tested alkaloids was significantly increased when tested as binary or TAEs indicating a kind of synergism among these alkaloids.

### 3.5. Sublethal Effects of Total Alkaloid Extracts (TAEs) on Larval Development

For the newly emerged second-instar larvae exposure to sublethal concentrations of TAEs at 48 h post-treatment, the obtained results revealed that TAEs at LC_10_ and LC_30_ concentrations exhibited sublethal effects on larval development time, as well as pupation and emergence rate (Table 2). The developmental time of the larvae stage was 183.9 h and 201.3 h days significantly longer in the LC_10_ (*p* < 0.05) and LC_30_ (*p* < 0.01) treatments, respectively than in the control. The pupa time was also significantly prolonged at 67.2 h and 83.5 h in LC_10_ and LC_30_ treatments, respectively. Additionally, the pupation and emergence rates of LC_30_ treatment were more significantly decreased than the control (*p* < 0.05). The results indicated that sublethal concentrations of TAEs could delay the development of *A. albopictus* larvae and decrease the reproductive potential of *A. albopictus* individuals.

## 4. Discussion

With the increasing demand for more eco-friendly products for mosquito control, plants may be valuable sources for mosquito control products. Plants can produce a vast repository of secondary compounds with a wide range of biological activities such as insecticidal activity [8]. Plant-derived alkaloids, either as plant-derived insecticides or as pure compounds, provide unlimited opportunities for new and selective pesticide discoveries because of the varied insecticidal active targets and unmatched availability of chemical diversity [9]. In this study, total alkaloids and four isolated alkaloids of *P. harmala* exhibited considerable larvicidal activity against *A. albopictus* larvae in a dose-dependent manner. Especially, the TAEs exhibited strong larvicidal activity against all instar larvae of *A. albopictus* at 48 h post-treatment (Figure 1a). Comparing the achieved LC_50_values, the larvicidal activity of the tested alkaloids in a descending order was TAEs > harmaline > harmine > harmalol (Figure 1b). Similarly, Shang et al. [22] (2016) observed the acaricidal activity of the total alkaloids from *P. harmala* on *Psoroptes cuniculi*. The authors also found that extracts of three bioactive alkaloids exhibited excellent acaricidal activity, as confirmed by our previous study [18] as well as this one. The authors Miao et al. [24] (2020) and Abbassi et al. [21] (2003) also demonstrated some of our observations. For example, the authors observed a toxic effect in harmaline and the extract of *P. harmala* seeds on the mortality of *Caenorhabditis elegans* and *Spodoptera exigua*. Therefore, extensive bioassays on larvicidal properties of the alkaloid components are needed to reveal possible synergisms, especially in those cases where the total alkaloids are more active than their individual main components such as harmaline and harmine.

It is well known that secondary metabolites always exist in plants in the form of simple or complex mixtures; there are thus many hypotheses about the so-called phytochemical redundancy [38]. Accordingly, evaluating for the pesticidal activity of specific phytochemicals must be supplemented by the appropriate testing of relevant mixtures and the whole extracts [31,32,33]. In this study, doses matching the estimated LC_25_ were used to evaluate the biological activity of simple or complex mixtures of the tested alkaloids; results showed that the action of a single alkaloid usually resulted in evidently lower larval mortality than the expected 25%. Despite that, when included in binary mixtures, some combinations resulted in a larval mortality greater than 70% (Table 1). This phenomenon was observed in binary mixtures of harmaline/harmine or harmaline/harmalol, indicating a kind of synergism among these compounds. Rizwan-ul-Haq et al. [33] (2009) obtained similar results regarding the two alkaloids harmaline and ricinine from isolated the seeds of *P. harmala* and *Ricinus communis*, respectively. They found that the insecticidal activity of these alkaloids on *Spodoptera exigua* larvae was significant when treated alone, while a mixture of both alkaloids at the same dose was shown to be more effective. Another study also found that the antibacterial and antifungal activities of isolated alkaloids (i.e., harmine, harmaline, and harmalol or total alkaloids) was significantly increased, when tested as a binary or total alkaloidal mixture, indicating a kind of synergistic effect among these compounds [32]. Synergism between different chemical components in plants has been documented, in which the toxicity or other active effects of one compound are significantly increased in the presence of other compounds [31]. The optimal efficacy of a medicinal plant may not be due to one major active ingredient, but to the combined effect of different compounds in the plant [8] (Dinesh, et al., 2014). Due to such responses commonly occurring in the plant-derived extracts, it is thus important to select an active synergistic mixture with the insecticidal properties. Thus, it could be concluded that the total alkaloids or binary mixture from of *P. harmala* have the potential for new and selective insecticidal agents for mosquito vector control. However, the mechanism behind the synergism among these beta-carboline alkaloids is still to be further explored.

Recently, plant-derived biopesticides have been used against many insect pests, especially vector mosquitoes, because plant-derived compounds (e.g., alkaloids, essential oils, and terpenoids) are safer to use and have a wide range of sublethal effects such as biochemical metabolism abnormality, growth and/or reproduction inhibition, adult repellent, and oviposition deterrent [9]. In the present study, the TAEs could significantly delay the development of *A. albopictus* larvae and pupae, when treated with sublethal concentrations (LC_10_ and LC_25_). In addition, the pupation rate and emergence rate of the surviving individuals also decreased significantly under LC_25_ exposure dose (Table 2). Our previous research found that total alkaloids and harmalin from *P. harmala* could significantly inhibit the absorption of Na^+^, K^+^, and glucose by Sf9 cells, as well as the number of blood cells of *S. frugiperda* larvae when exposed to LC_50_ dose [20] (Li et al., 2016). Jbilou et al. (2008) [26] found that *P. harmala* extracts exhibited many sublethal effects on *Tribolium castaneum*, including α-amylase activity, larval development, and progeny production. Similarly, another two studies also observed sublethal effects of harmalin on *Spodoptera exigua* and *Plodia interpunctella* larvae, which involved nutrient metabolism, a-amylase activity, and larval growth [23,33], as confirmed by our previous study [19]. Further analysis concluded that sublethal effects on insect growth may result from disruptions in the development of neural tissues by *P. harmala* alkaloids, especially harmaline and harmine [39,40]. Another reason may be that energy intake is limited or energy is used for detoxification, which is not enough for growth and development, as confirmed by previous studies [29]. However, further studies need to be conducted on the synergistic mechanism and the long-term sublethal effects on generations.

## 5. Conclusions

In this study, the total alkaloid extracts (TAEs) and beta-carboline alkaloids (harmaline, harmine, harmalol, and harman) from *Peganum harmala* seeds were isolated and tested in this bioassay. The results revealed considerable toxicity of the TAEs and isolated beta-carboline alkaloids against *A. albopictus* larvae. Especially, all compounds (such as TAEs, harmaline, and harmine) showed their synergistic effects, exceeding the toxicity of each compound alone. Interestingly, the obtained data further showed that the TAEs at sublethal doses (LC_10_ and LC_25_) could significantly delay the larval development, and decrease the pupation and emergence rates of *A. albopictus*. This phenomenon could be helpful in order to develop more effective control strategies for different notorious vector mosquitoes.

## Figures and Tables

**Table 1:** Toxicity of total alkaloid extracts (TAEs) and isolated alkaloids from *Peganum harmala* seeds and their binary mixtures prepared as LC_25_ combinations against second-instar larvae of *Aedes albopictus*.

Treatments	Mean Mortality (%) ± SE		Co-Toxicity Coefficient		Type of Effect	
	24 h	48 h	24 h	48 h	24 h	48 h
Harmaline (A)	22.3 ± 1.1 ^d^	29.7 ± 1.3 ^d^	-	-	-	-
Harmine (B)	19.8 ± 0.9 ^d^	23.6 ± 1.1 ^d^	-	-	-	-
Harmalol (C)	18.6 ± 1.2 ^d^	22.3 ± 1.4 ^d^	-	-	-	-
Harmane (D)	17.9 ± 1.3 ^d^	21.5 ± 1.2 ^d^	-	-	-	-
A + B	59.2 ± 2.1 ^b^	79.3 ± 2.1 ^a^	+141	+149	Synergism	Synergism
A + C	56.1 ± 1.9 ^b^	72.5 ± 2.4 ^b^	+137	+139	Synergism	Synergism
A + D	55.6 ± 2.2 ^b^	69.5 ± 1.9 ^b^	+138	+136	Synergism	Synergism
B + C	52.3 ± 1.8 ^c^	60.2 ± 2.1 ^c^	+136	+131	Synergism	Synergism
B + D	50.4 ± 2.0 ^c^	54.9 ± 1.7 ^c^	+134	+122	Synergism	Synergism
C + D	48.3 ± 1.6 ^c^	51.9 ± 1.9 ^c^	+132	+119	Synergism	Synergism
TAEs	65.2 ± 2.4 ^a^	83.1 ± 2.6 ^a^	-	-	Synergism	Synergism

Co-toxicity coefficient (CTC) = [(*A* + *B*)_exper_/(*A* + *B*)_theor_] × 100 [37]. Each datum represents the mean of three replicates. In the same column, means followed by the same letters are not significantly different (*p* > 0.05).

**Table 2:** Sublethal effects of total alkaloid extracts (TAEs) from *Peganum harmala* seeds on the second-instar larval development.

Treatment	Number of Larvae Tested	Larvae Grow Time/h(Mean ± SE)	Pupa Time/h (Mean ± SE)	Pupation Rate/% (Mean ± SE)	Emergence Rate/% (Mean ± SE)
Control	210	156.8 ± 6.2 ^c^	55.3 ± 6.5 ^c^	96.8 ± 2.1 ^a^	94.1 ± 3.9 ^a^
LC_10_	210	183.9 ± 8.2 ^b^	67.2 ± 7.3 ^b^	89.4 ± 5.6 ^a^	87.3 ± 6.3 ^a^
LC_30_	240	201.3 ± 9.3 ^a^	83.5 ± 8.1 ^a^	74.5 ± 6.2 ^b^	65.4 ± 5.1 ^b^

Each datum represents the mean of three replicates. Mean in the same column followed by different letters are significantly different (*p* < 0.05).

## Data Availability

Not applicable.

## Abbreviations

TAEs, total alkaloid extracts of *Peganum harmala* seeds; CTC, co-toxicity coefficient; LC_50_, lethal concentration to 50% of the test population; LC_25_, lethal concentration to 25% of the test population; HPLC, high-performance liquid chromatography.
